# Structural insights into subtype-specific agonist recognition by sphingosine-1-phosphate receptors

**DOI:** 10.1371/journal.pbio.3003381

**Published:** 2026-04-10

**Authors:** Leiye Yu, Haizhan Jiao, Bin Pang, Rujuan Ti, Bing Gan, Zhaoyang Qin, Jinxin Wang, Lizhe Zhu, Hongli Hu, Ruobing Ren

**Affiliations:** 1 Shanghai Key Laboratory of Metabolic Remodeling and Health, Institute of Metabolism and Integrative Biology, Shanghai Xuhui Central Hospital, Zhongshan-Xuhui Hospital, Fudan University, Shanghai, China; 2 Department of Otorhinolaryngology Head and Neck Surgery, Shanghai Sixth People’s Hospital Affiliated to Shanghai Jiao Tong University School of Medicine, Shanghai, China; 3 Kobilka Institute of Innovative Drug Discovery, School of Life and Health Sciences, The Chinese University of Hong Kong (Shenzhen), Shenzhen, China; 4 Warshal Institute of Computational Biology, School of Life and Health Sciences, The Chinese University of Hong Kong (Shenzhen), Shenzhen, China; 5 School of Pharmacy, Second Military Medical University, Shanghai, China; Zhejiang University School of Medicine, CHINA

## Abstract

Sphingosine-1-phosphate (S1P), a key metabolite of sphingolipids, plays crucial roles in a wide range of physiological and pathological processes. S1P primarily exerts its functions by binding to G protein-coupled sphingosine-1-phosphate receptors (S1PRs), which comprise five subtypes (S1PR1-5) in humans, thereby activating these receptors and their downstream signaling pathways. Understanding the molecular determinants that govern agonist selectivity among different S1PR subtypes is vital for the rational and precise development of targeted therapeutic agents. Here, four cryo-electron microscopy structures of agonist-bound S1PR1-Gi1 complexes are reported. Through an integrated approach combining structural analysis, molecular dynamics simulations, and pharmacological assays, the molecular basis for the selectivity of CYM5442, HY-X-1011, Ponesimod, and SAR247799 toward S1PR1 over S1PR2-S1PR5 is uncovered. Nonconserved residues within the ligand-binding pocket and at the Gi1-protein interface contribute to S1PR1 selectivity by these agonists. A distinct agonist binding orientation toward transmembrane helices 5-7, combined with branched substituents that increase the agonist’s molecular width, results in steric clashes with residues in S1PR3. Additionally, branched moieties located at the tail portions of the agonist restrict its deep insertion into the binding pocket of both S1PR3 and S1PR5. These structural features collectively enhance its selectivity for S1PR1 over S1PR3 and S1PR5. Furthermore, polar interactions with conserved polar residues in the top region of the binding pocket also influence agonist selectivity. Besides, the relatively broad molecular width of the agonist sterically hinders its binding into S1PR2 and S1PR4 pocket by nonconserved residue pairs bearing bulky side chains. These findings establish a structural framework for the rational design of next-generation S1PR1 highly selective agonists with improved therapeutic potential.

## Abstract 2

Sphingosine-1-phosphate receptors (S1PRs) play complex roles in several pathological processes, which has led to the development of pharmacological modulators. This study solves the cryo-EM structures of four agonist-bound S1PR1-Gi1 complexes and reveals the structural determinants underlying agonist selectivity among different subtypes.

## 1. Introduction

Sphingosine-1-phosphate (S1P), a key metabolite of sphingolipids, plays crucial roles in a wide range of physiological processes, such as lymphocyte trafficking and cardiovascular development. S1P is generated through the phosphorylation of sphingosine, a backbone structural component of all sphingolipids, and can be exported from cells via specific membrane transporters [1–4]. S1P primarily exerts its biological functions by binding to G protein-coupled sphingosine-1-phosphate receptors (S1PRs), which consist of five subtypes (S1PR1-5), thereby activating these receptors and triggering various downstream signaling pathways [5]. S1PRs belong to the class A GPCR (G protein-coupled receptor) superfamily. Activation of GPCRs involves significant conformational rearrangements on the cytoplasmic side, particularly a large outward movement of transmembrane helix 6 (TM6), accompanied by reorganization of other helices, which creates an intracellular pocket that can engage different G proteins (Gi/o, Gs, Gq, and G12/13), GRKs (G protein-coupled receptor kinases), and arrestins to form functional signaling complexes [6]. The S1PR subtypes activate distinct yet partially overlapping G protein-mediated signaling cascades, thus orchestrating diverse cellular responses such as proliferation, apoptosis, adhesion, and migration. Specifically, S1PR1 couples exclusively with Gi/o (the alpha subunit of heterotrimeric G proteins), S1PR2-3 interact with Gi/o, G12/13, and Gq, while S1PR4-5 bind to Gi/o and G12/13 [5].

S1PR1 is highly expressed in endothelial cells and adipocytes, with lower expression levels observed in various immune cell populations. Upon activation by S1P, S1PR1 engages Gi proteins, leading to subsequent activation of Rac1 signaling, which promotes the formation of tight junctions between endothelial cells and helps maintain the integrity and normal function of vascular and lymphatic endothelial barriers [7]. In vascular endothelial cells (VECs), S1PR1 signaling not only stabilizes adherens junctions but also enhances nitric oxide (NO) production via endothelial nitric oxide synthase (eNOS), which is essential for regulating blood flow and pressure [8]. Moreover, S1PR1 plays a pivotal role in lymphocyte egress from the thymus and secondary lymphoid organs, a process that relies on a concentration gradient of S1P-high in plasma (~1 μM) and lymph (~100 nM), compared to much lower levels in interstitial fluids [9–13]. Additionally, S1PR1 is expressed in brain endothelial cells, astrocytes, glial cells, and leukocytes, where it contributes significantly to the proper functioning of the central nervous system (CNS).

S1PRs play complex roles in multiple pathological processes, including cardiovascular, autoimmune, inflammatory, neurological, oncologic, hearing loss, and fibrotic diseases [14]. Consequently, the therapeutic potential of S1PR1 modulation has been extensively investigated, leading to the development of numerous S1PR1 modulators, several of which are clinically approved pharmacological agents for treating various conditions such as multiple sclerosis (MS) and inflammatory bowel disease (IBD) [15–18]. MS is a chronic neurodegenerative disorder characterized by persistent inflammation within the CNS, resulting in neuronal demyelination and subsequent functional impairments. Fingolimod (FTY720) was the first FDA-approved drug specifically targeting S1PR1 for the treatment of MS [16]. Fingolimod undergoes phosphorylation in vivo to form phosphorylated Fingolimod (FTY720-P). Upon binding to S1PR1, FTY720-P activates S1PR1 and promotes β-arrestin recruitment, leading to β-arrestin-mediated internalization of S1PR1. This results in a reduction of S1PR1 on the plasma membrane, thereby decreasing the circulation and CNS infiltration of immune cells, which suppresses inflammation and disease progression. However, patients treated with Fingolimod may experience adverse events such as relapse of multiple sclerosis, decreased lymphocyte count, fatigue, and headache [19]. Furthermore, FTY720-P also activates all five S1PR subtypes and engages G protein-dependent signaling pathways that can promote immune cell migration [20]. Additionally, its long half-life contributes to further side effects [21]. In recent years, the FDA has approved three second-generation S1PR-targeted drugs—Siponimod, Ozanimod, and Ponesimod—for the treatment of MS [22–24]. Although these newer agents exhibit improved receptor selectivity, they still carry risks of side effects such as bradycardia. Siponimod and Ozanimod selectively activate both S1PR1 and S1PR5 with similar potency, with Siponimod showing approximately a thousand-fold lower potency toward S1PR3 and S1PR4 compared to S1PR1 and S1PR5 [25,26]. Ponesimod strongly activates S1PR1 but exhibits lower affinity for S1PR3 and S1PR5 [27]. The activation of multiple S1PR subtypes may lead to varied physiological effects and increase the likelihood of certain adverse reactions, such as bradycardia [28].

It is noteworthy that current therapeutics predominantly target S1PR1 as their primary mechanism of action. However, due to the high sequence and structural similarity among the five S1PR subtypes, existing modulators often display cross-reactivity across multiple receptor subtypes, activating diverse signaling pathways. This phenomenon not only complicates therapeutic outcomes but also contributes to the potential side effects associated with these drugs. Therefore, the development of highly subtype-selective S1PR modulators remains a significant challenge. Understanding the molecular determinants that govern agonist selectivity among S1PR subtypes is crucial for the rational and precise design of targeted modulators. While multiple structures of S1PRs bound to various modulators have been reported previously [29–37], the mechanisms underlying agonist selectivity among different S1PR subtypes remain insufficiently understood. In this study, we determined four agonist-bound S1PR1-Gi1 complex structures. Through integrated structural analysis, mutagenesis combined with Gi1 coupling assays, and molecular dynamics simulations, we uncovered the molecular basis for the preferential selectivity of agonists toward S1PR1 over S1PR2-S1PR5. These findings provide a foundation for the rational design and development of highly selective S1PR1 modulators.

## 2. Results

### 2.1 S1PRs subtype selectivity of four S1PR1 agonists

**Fig 1:**
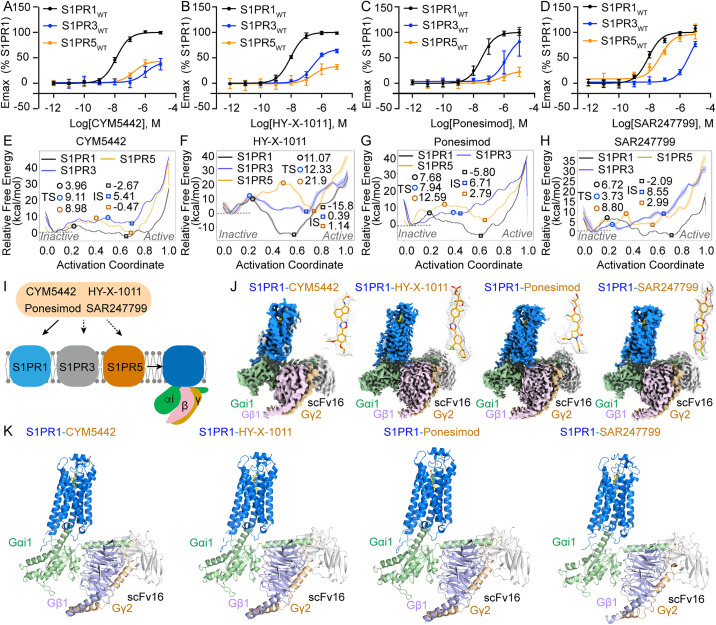
Structures of four S1PR1-Gi1 complexes bound with CYM5442, HY-X-1011, Ponesimod, or SAR247799. **(A–D)** Activation of S1PR1, S1PR3, and S1PR5 by CYM5442, HY-X-1011, Ponesimod, or SAR247799, as measured via the BRET-based Gi1 dissociation assay. Data are presented as mean ± SD; *n* = 3. **(E–H)** Free energy profiles along the activation pathways of S1PR1 (black), S1PR3 (blue), and S1PR5 (wheat) induced by CYM5442, HY-X-1011, Ponesimod, or SAR247799. The profiles are plotted as lines. TS: transition state; IS: intermediate state, structurally resembling the fully active conformation. The free energy differences between the IS/TS states and the inactive state are calculated and labeled using circles (TS) and squares (IS), respectively. **(I)** Schematic illustration showing the activation of S1PR1, S1PR3, and S1PR5 by the four agonists—CYM5442, HY-X-1011, Ponesimod, and SAR247799. Solid arrows indicate strong activation, while dashed arrows represent weak activation. **(J)** Cryo-EM density maps of S1PR1-Gi1 complexes bound with CYM5442, HY-X-1011, Ponesimod, or SAR247799. Ligands (yellow) are displayed within the density maps (gray mesh) on the right side of the models and are represented in stick form. **(K)** Structural cartoon models of human S1PR1 in complex with Gi1, scFv16, and each of the four agonists: CYM5442, HY-X-1011, Ponesimod, or SAR247799. In panels (J and K) S1PR1 is colored marine, Gαi1 pale green, Gβ1 light blue, Gγ2 wheat, scFv16 gray, and agonists yellow. The underlying data for Fig 1A–1H can be found in S1 Data.

Previously, several agonists—CYM5442, HY-X-1011, Ponesimod, and SAR247799—were reported to exhibit improved selectivity for S1PR1 over S1PR2-S1PR5 (S1A–S1D Fig) [38–41]. Although the activation of S1PR by these four agonists has been characterized previously using various experimental approaches, we employed a standardized bioluminescence resonance energy transfer (BRET)-based assay to systematically evaluate their activation profiles across S1PR1-S1PR5. This method enables quantitative assessment of Gi1 protein dissociation kinetics and facilitates direct comparison of four agonists pharmacological properties. The BRET assay results demonstrated that all four agonists exhibit greater potency and efficacy for S1PR1 compared to S1PR3 and S1PR5, with EC_50_ values differing by one to two orders of magnitude (Fig 1A–1D). In contrast, S1P exhibits comparable potency across S1PR1, S1PR3, and S1PR5 (S1E Fig). Furthermore, no activation of S1PR2 and S1PR4 was detected in response to any of the four agonists in the BRET-based Gi1 dissociation assay, unlike the robust activation observed with S1P (S1F–S1G Fig).

A traveling-salesman based automated path searching (TAPS) molecular dynamics simulations method was employed to predict S1PR1/3/5 activation by agonists and successfully identified a novel S1PR1 agonist (HY-X-1011) with enhanced selectivity for S1PR1 over S1PR3 and S1PR5 [42]. In this study, we applied the same TAPS-based molecular dynamics method to explore the minimum free energy path (MFEP) during the transition of S1PR1/3/5 from an inactive to an active conformation induced by CYM5442, HY-X-1011, Ponesimod, and SAR247799 (details provided in the Methods section). The activation potential of each ligand can be quantified by the sum of the barrier height ΔG_T_ (the Gibbs free energy difference between the inactive state and the transition state) and the stability of the intermediate state relative to the inactive state ΔG_I_ (the Gibbs free energy difference between the inactive state and the intermediate state; the intermediate state closely resembles the fully active structure) [41]. The calculated free energy distributions (ΔG_T_ + ΔG_I_) along the MFEP of the four distinct ligands for three S1PR subtypes showed strong agreement with the BRET measurements of Gi1 protein dissociation (Fig 1E–1H).

### 2.2 Overall structures of agonist-bound S1PR1-Gi1 complexes

However, the lack of comprehensive structural, computational, and pharmacological profiling has limited our understanding of the mechanisms underlying receptor subtype selectivity (Fig 1I). To investigate and clarify the basis of this selectivity, we determined four Gi1-coupled S1PR1 structures bound to CYM5442, HY-X-1011, Ponesimod, or SAR247799 using single-particle cryo-electron microscopy (cryo-EM). The four complexes were successfully assembled following previously established protocols (S2A–S2D Fig) [33]. Data collection and analysis yielded four density maps of the S1PR1-Gi1 complex bound to each respective agonist at overall resolutions of 3.69 Å, 2.79 Å, 2.79 Å, and 2.97 Å, respectively, enabling structural model building (Figs 1J–1K, S3A–S3E and S1 Table). Most residues with bulky side chains in the transmembrane helices (TM) 2-7 of the receptor can be identified. However, the density for TM1 is noticeably weaker than that of TM2-TM7 across all four maps (S3F–S3I Fig). Importantly, densities in the ligand-binding pocket for each of the four agonists were observed in their respective maps, enabling the determination of the ligand’s overall orientation, and a detailed characterization of the binding sites (Figs 1J and S4).

### 2.3 Agonist-induced activation of S1PR1

**Fig 2:**
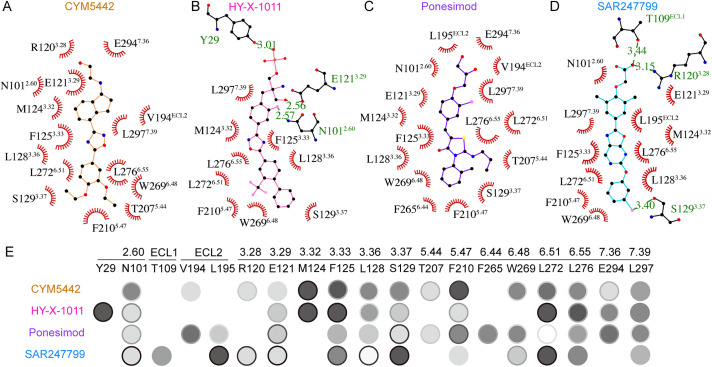
Interaction networks drive S1PR1 activation. **(A–D)** Residues within the ligand-binding pocket that interact with CYM5442 (A), HY-X-1011 (B), Ponesimod (C), or SAR247799 (D). Hydrogen bonds are indicated by green lines, with corresponding distances labeled in angstroms (Å). **(E)** The effect of pocket amino acid mutations on receptor activation induced by CYM5442, HY-X-1011, Ponesimod, or SAR247799, as measured by the BRET-based Gi1 dissociation assay. In this assay, residues were assessed using alanine scanning mutagenesis to evaluate their effects on the Gi1 signaling response. The background color of the circles indicates potency, while the border color represents efficiency. A darker circle background corresponds to a greater reduction in potency, and a darker circle border reflects a more pronounced decrease in efficacy upon mutation. Blank indicates that the effect of the amino acid mutation on receptor activation and Gi1 signaling has not been evaluated. Detailed quantitative data are provided in S2 Table of the Supporting Information. The underlying data for Fig 2E can be found in S2 Data.

In these Gi1-coupled S1PR1 structures, four agonists adopt extended conformations within the orthosteric binding pocket of S1PR1, exhibiting length-dependent variations in polar interactions in the top region of the pocket, while maintaining similar hydrophobic contacts in the middle and bottom regions (Fig 2A–2D). Systematic alanine scanning mutagenesis confirmed the significant roles of most selected residues involved in agonist interaction, as revealed by Gi1 dissociation BRET assays (Fig 2E and S2 Table). Alanine substitution of polar residues, including Y29, N101^2.60^, R120^3.28^, and E121^3.29^, has been shown to significantly reduce S1PR1 activation by both S1P and FTY720P [33]. These residues which interact with the head groups of CYM5442, HY-X-1011, Ponesimod, or SAR247799 also affect the potency or efficacy of agonist-induced S1PR1 activation to varying degrees. Notably, most residues in the middle and bottom regions of the pocket strongly influence the potency or efficacy of all four agonists, such as F125^3.33^, L128^3.36^, L276^6.55^, and L297^7.39^. The key residues critical for CYM5442, HY-X-1011, Ponesimod, or SAR247799 induced S1PR1 activation are predominantly located in transmembrane segments TM3, TM5, TM6, and TM7 with their densities clearly discernible (S4 Fig). These results suggest that, in comparison to the head groups, the hydrophobic tails of these agonists exhibit stronger interactions with TM3, TM5, TM6, and TM7, playing more critical roles in activating S1PR1 and initiating downstream Gi1 signaling.

A comparison of conformational changes between agonist-bound active S1PR1 structures and the antagonist ML-056-bound inactive structure provides key mechanistic insights into receptor activation [29]. All active structures display a characteristic outward movement of TM6 by ~11.5 Å, which facilitates G protein coupling (S5A–S5D Fig). Agonist binding induces a conserved reorientation of side chains in the hydrophobic residues L128^3.36^, F210^5.47^, F273^6.52^, W269^6.48^, and L297^7.39^ (S5E–S5H Fig), with F210⁵^.^⁴⁷ undergoing a rotamer switch that drives TM6 displacement, accompanied by rotation of F273^6.52^. Furthermore, class A GPCR activation-related motifs in S1PR1—including E^3.49^R^3.50^Y^3.51^, P^5.50^I^3.40^F^6.44^, and N^7.49^P^7.50^xxY^7.53^—are largely conserved [43]. These motifs (E141^3.49^R142^3.50^Y143^3.51^, L213^5.50^V132^3.40^F265^6.44^, and N307^7.49^P308^7.50^xxY311^7.53^) exhibit significant conformational changes across all four activated receptor structures (S5I–S5T Fig), underscoring the evolutionary conservation of the S1PR activation mechanism.

### 2.4 Role of nonconserved residues in S1PR1 selectivity

**Fig 3:**
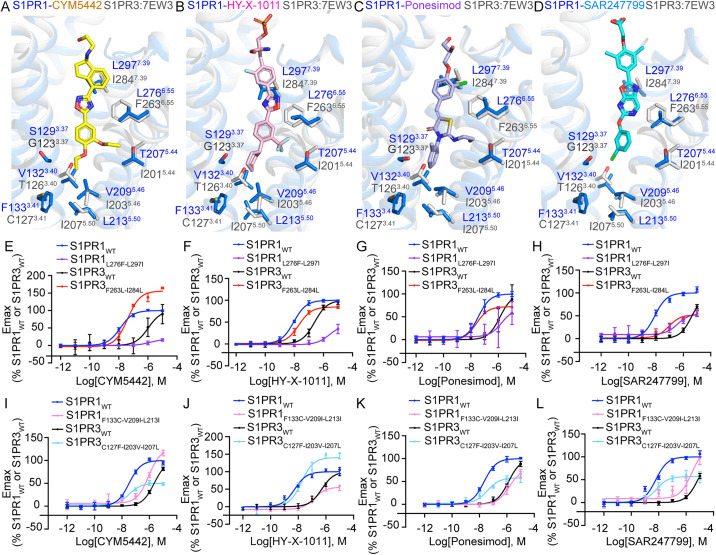
Nonconserved residues influence S1PR1 and S1PR3 selectivity. **(A–D)** Nonconserved residues in the ligand-binding pocket of S1PR1 and S1PR3. CYM5442-, HY-X-1011-, Ponesimod-, or SAR247799-bound S1PR1 are superimposed with S1PR3 (PDB: 7EW3) in panels (A–D), respectively. Residues of S1PR1 (marine) and S1PR3 (gray) are shown as sticks. Molecules are shown as sticks and colored yellow (CYM5442), pink (HY-X-1011), light blue (Ponesimod), and cyan (SAR247799). **(E–L)** BRET-based Gi1 dissociation assays showing activation of swapped mutants of S1PR1 and S1PR3 by the four agonists. Data are presented as mean ± SD; *n* = 3. The underlying data for Fig 3E–3L can be found in S3 Data.

Although most residues that interact with the four agonists are conserved across S1PR1, S1PR3, and S1PR5, we hypothesize that nonconserved residues within the ligand-binding pocket contribute to receptor subtype selectivity. Initially, we identified nonconserved residues in the ligand-binding pockets of S1PR1 and S1PR3 (Fig 3A–3D), which are located in the middle and bottom regions of the binding cavity. Two previously reported key residues—L276^6.55^ and L297^7.39^ in S1PR1, corresponding to F263^6.55^ and I284^7.39^ in S1PR3—are known to influence the selectivity of Siponimod and CBP-307 for S1PR1 and S1PR3, respectively. Functional characterization of reciprocal mutants confirmed the importance of these two residue pairs. The L276^6.55^F-L297^7.39^I double mutant of S1PR1 exhibited reduced agonist potency and efficacy, similar to or weaker than that of wild-type (WT) S1PR3. Conversely, introducing the F263^6.55^L-I284^7.39^L mutations into S1PR3 enhanced the potency of CYM5442, HY-X-1011, and Ponesimod to levels approaching those observed for S1PR1, although SAR247799 remained tenfold less potent (Fig 3E–3H and S3 Table). Additionally, residues S129^3.37^, T207^5.44^, and V132^3.40^ in S1PR1 correspond to G123^3.37^, I201^5.44^, and T126^3.40^ in S1PR3, and all contact the tail portions of the four agonists. Therefore, we examined the effects of mutating these three residue pairs—S129^3.37^G/G123^3.37^S, T207^5.44^I/I201^5.44^T, and V132^3.40^T/T126^3.40^V—on receptor activation. These mutants displayed partial exchange effects in response to Ponesimod and SAR247799, except for V132^3.40^T in S1PR1 and T126^3.40^V in S1PR3, which had no observable impact (S6A–S6F Fig). Three nonconserved residues—F133^3.41^, V209^5.46^, and L213^5.50^—located at the bottom of the S1PR1 pocket show distinct interaction patterns. Among them, V209^5.46^ interact with the tail ends of CYM5442, HY-X-1011, and Ponesimod. In contrast, F133^3.41^, and L213^5.50^ do not exhibit direct interactions with any of the four agonists in the determined structure. Intriguingly, results from the Gi dissociation assay showed that triple mutants F133^3.41^C-V209^5.46^I-L213^5.50^I (S1PR1) and C127^3.41^F-I203^5.46^V-I207^5.50^L (S1PR3) exhibited exchanged activation potencies when stimulated by CYM5442, HY-X-1011, Ponesimod, and SAR247799 (Fig 3I–3L and S3 Table).

**Fig 4:**
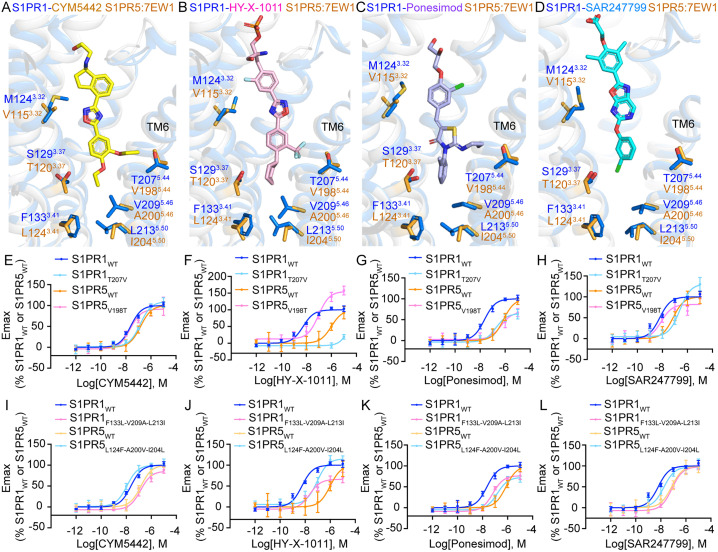
Nonconserved residues influence S1PR1 and S1PR5 selectivity. **(A–D)** Nonconserved residues in the ligand-binding pocket of S1PR1 and S1PR5. CYM5442-, HY-X-1011-, Ponesimod-, or SAR247799-bound S1PR1 are superimposed with S1PR5(PDB: 7EW1). Residues of S1PR1 (marine) and S1PR5 (orange) are shown as sticks. Molecules are shown as sticks and colored yellow (CYM5442), pink (HY-X-1011), light blue (Ponesimod), and cyan (SAR247799). **(E–L)** BRET-based Gi dissociation assays showing activation of S1PR1 and S1PR5 swapped mutants by the four agonists. Data are presented as mean ± SD; *n* = 3. The underlying data for Fig 4E–4L can be found in S4 Data.

Similarly, we also identified several residues predominantly clustered in the central and bottom regions of the binding pockets of S1PR1 and S1PR5 (Fig 4A–4D). Among them, M124^3.32^ and S129^3.37^ (S1PR1), corresponding to V115^3.32^ and T120^3.37^ (S1PR5), exhibit close contacts with four agonists. However, these reciprocal mutants exhibit varying effects on the activation of S1PR1 and S1PR5 by the four agonists (S7A–S7H Fig). We further analyzed T207^5.44^ and V198^5.44^, which are located between TM5 and TM6, near the branched moieties at the tail portions of CYM5442, HY-X-1011, and Ponesimod. The T207^5.44^V mutation in S1PR1 results in reduced potency, similar to the activation of S1PR5 by the four agonists, whereas the V198^5.44^T mutation in S1PR5 leads to increased potency, resembling the activation of S1PR1 by CYM5442, HY-X-1011, and SAR247799 (Fig 4E–4H). Additionally, three residues—F133^3.41^, V209^5.46^, and L213^5.50^—at the bottom of the S1PR1 pocket are also not conserved in S1PR5 (L124^3.41^, A200^5.46^, and I204^5.50^) (Fig 4A–4D). Reciprocal triple mutations—F133^3.41^L-V209^5.46^A-L213^5.50^I in S1PR1 and L127^3.41^F-A203^5.46^V-I207^5.50^L in S1PR5—resulted in fully swapped activation profiles for CYM5442 and SAR247799, and showed significant exchange in activation effects for Ponesimod and HY-X-1011, except for L127^3.41^F-A203^5.46^V-I207^5.50^L in S1PR5 in response to Ponesimod (Fig 4I–4L). Collectively, our findings systematically reveal nonconserved residue pairs within the ligand-binding pocket that contribute to S1PR1 selectivity by the four agonists.

Most nonconserved residues within the ligand-binding pocket influence receptor subtype selectivity through direct contact with agonists. However, nonconserved residues at positions 3.41, 5.46, and 5.50—two of which do not directly interact with the four agonists in our determined structures—nonetheless play significant roles in determining selectivity for S1PR1, S1PR3, and S1PR5, as revealed by above analyses. To further understand the structural basis underlying the effects of these nonconserved residues at positions 3.41, 5.46, and 5.50 on subtype selectivity, we conducted additional structural analysis.

We observed that residues F/C/L^3.41^, V/I/A^5.46^, and L/I/I^5.50^ are located at the bottom of the orthosteric pockets in S1PR1, S1PR3, and S1PR5 (Figs 3A–3D and 4A–4D). Moreover, compared to the inactive structure of S1PR1, F133^3.41^, V209^5.46^, and L213^5.50^ move closer to each other in the active structure, leading to stronger interactions between F133^3.41^ and L213^5.50^ and enhancing contacts between TM3 and TM5 (S8A–S8E Fig). However, the shorter or smaller side chains of the corresponding residues in S1PR3 (C127^3.41^ and I207^5.50^) and S1PR5 (L124^3.41^ and I204^5.50^) weaken these interactions in those subtypes. Additionally, L/I/I^5.50^ is part of the conserved Class A GPCR activation-related motif P^5.50^I^3.40^F^6.44^, which influence receptor activation.

Furthermore, we found that R78^2.37^ in S1PR1, located at the Gi-binding interface and interacting with D350 on the α5 helix of Gi1, is not conserved in S1PR5 (A69^2.37^) and S1PR3 (N72^2.37^) (S9A Fig). Gi1 dissociation assays results of swapped mutants confirmed that R78^2.37^ in S1PR1 and A69^2.37^ in S1PR5 also influence the selectivity of S1PR1 and S1PR5 by four agonists (S9B–S9E Fig).

Notably, the four agonists discussed above do not activate S1PR2 or S1PR4. This lack of activation is attributed to nonconserved amino acid residues in these two receptors. H271^7.31^ and F274^7.39^ in S1PR2, and L125^3.32^ and M289^7.35^ in S1PR4 with bulky side chains result in a significantly narrower cavity at the upper region of the ligand-binding pocket in both S1PR2 and S1PR4 (S10A–S10H Fig). Consequently, the above-mentioned agonists, due to their relatively broad molecular width, are sterically hindered from entering the binding pocket and reaching its deeper regions, thereby preventing effective binding and receptor activation. F274^7.39^ in S1PR2, and L125^3.32^ and M289^7.35^ in S1PR4 were also discussed in a previously report [44]. Moreover, the effects of other nonconserved amino acid residues on agonist selectivity for S1PR1 over S1PR2 and S1PR4 require further investigation.

### 2.5 Agonists structural features and binding modes for S1PR1 selectivity

**Fig 5:**
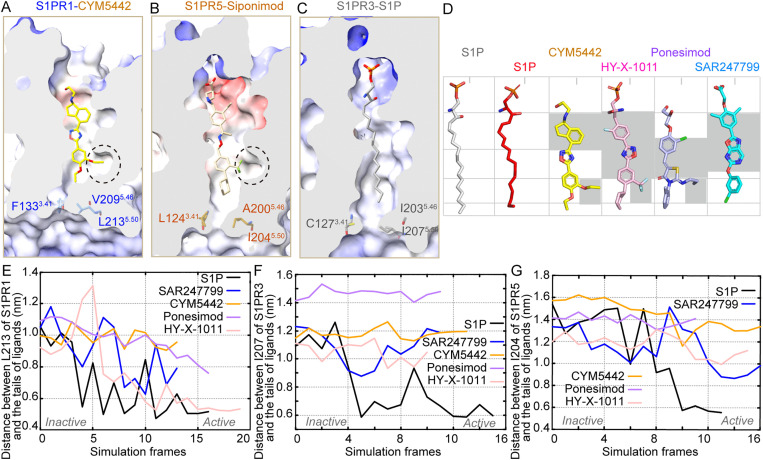
Ligand structural variations dictate their penetration depths into receptor pockets. **(A–C)** Ligand-binding pockets in S1PR1, S1PR3, and S1PR5. Subpockets at the bottom region of the ligand-binding pockets in S1PR1 and S1PR5 are indicated by black dashed lined circles. Three nonconserved residues are located at the bottom of the ligand-binding pockets in S1PR1, S1PR3, and S1PR5. Electrostatic potentials of the pocket and protein surface are displayed: red indicates negative charge, blue indicates positive charge, and white represents hydrophobic regions. The pocket-cutting surface is shown in gray, with all surfaces rendered as semitransparent. Structures include S1PR1 bound to CYM5442 (A), S1PR5 bound to Siponimod (PDB: 7EW1) (B), and S1PR3 bound to S1P (PDB: 7EW3) (C). The bottom portions of these pockets are open toward the intracellular side. Ligands and the three nonconserved residues at the base of each pocket are shown in stick representation. **(D)** Structural comparison of the five S1PR1 agonist molecules. Structures of five agonists binding within the ligand pocket are presented. S1P (gray) bound with S1PR3 (PDB: 7EW3), S1P (red) bound with S1PR1 (PDB: 7VIE), CYM5442 (yellow), HY-X-1011(pink), Ponesimod (lightblue), or SAR247799 (cyan) bound with S1PR1. The gray background highlights conserved features among four of the molecules. **(E–G)** Distances between the tails of CYM5442, HY-X-1011, Ponesimod, or SAR247799 and the Cα atom of residue ^5.50^ in S1PR1, S1PR3, and S1PR5 during receptor activation—from the inactive to active state—calculated using the TAPS method in molecular dynamics simulations. The underlying data for Fig 5E–5G can be found in S5 Data.

Notably, S1PR1 and S1PR5 contain a larger subpocket that accommodates branched groups present in the tail portions of CYM5442 (Fig 5A–5B). In contrast, S1PR3 features a smaller and shallower subpocket at the corresponding position (Fig 5C). Compared to S1P, the four synthetic agonists have a larger molecular width, and the branched functional moieties located at the tail portions of CYM5442, HY-X-1011, and Ponesimod appear to restrict their ability to penetrate deeper into the binding pocket during activation (Fig 5D). Thus, we hypothesize that S1P, which lacks the bulky branched substituent, can easily access the deeper regions of the receptor pocket during activation, thereby facilitating the activation of S1PR3 and S1PR5. Similarly, SAR247799, which lacks branched moieties at its tail portions, is more likely to penetrate deeply into the S1PR5 pocket. To test this hypothesis, we employed molecular dynamics simulations using our previously established TAPS method to examine agonist binding within the S1PR1/3/5 pockets during the transition from inactive to active receptor states. Distance measurements between the tail ends of the five agonists and residue 5.50 at the bottom of the S1PR1/3/5 pockets supported our hypothesis (Fig 5E–5G). Upon receptor activation, S1P penetrates deeply into the pockets of all three subtypes—S1PR1, S1PR3, and S1PR5—whereas CYM5442, HY-X-1011, and Ponesimod reach only relatively shallow positions in the pockets, obviously in S1PR3 and S1PR5. SAR247799 inserts more deeply into the S1PR5 pocket compared to CYM5442, HY-X-1011, and Ponesimod, consistent with its dual activity toward S1PR1 and S1PR5.

Although nonconserved residues among S1PR subtypes play crucial roles in ligand selectivity, the designed agonists exhibit greater selectivity for S1PR1 compared to S1P, which demonstrates broad affinity across all five S1PR subtypes. Beyond the above-mentioned structural differences between the four agonists and S1P, their distinct binding modes within the ligand-binding pocket may provide insights into subtype-specific signaling mechanisms mediated by these agonists. Therefore, we further analyzed and compared the binding modes of the four agonists with that of S1P.

**Fig 6:**
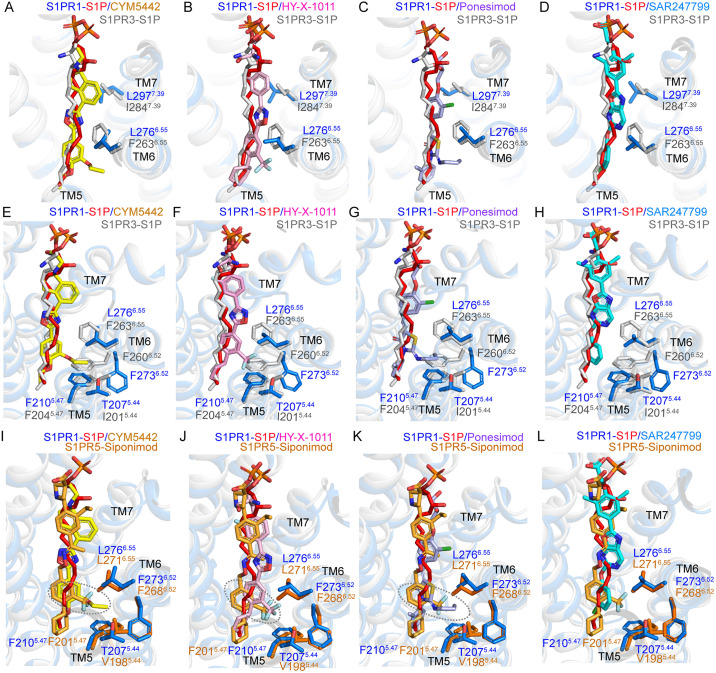
Comparison of agonists binding modes across S1PR1, S1PR3, and S1PR5. **(A–H)** Superimposition of CYM5442, HY-X-1011, Ponesimod, or SAR247799 bound to S1PR1 with S1P bound to S1PR1 (PDB: 7VIE) and S1PR3 (PDB: 7EW3). Residues of S1PR1 (marine) and S1PR3 (gray) are shown as sticks. Molecules are displayed as sticks colored as follows: yellow for CYM5442, pink for HY-X-1011, light blue for Ponesimod, cyan for SAR247799, red for S1P (from 7VIE), and gray for S1P (from 7EW3). **(I–L)** Superimposition of CYM5442, HY-X-1011, Ponesimod, SAR247799, and S1P in S1PR1 (PDB: 7VIE) with Siponimod in S1PR5 (PDB: 7EW1). Four residues of S1PR1 (marine) and S1PR5 (yellow-orange) are shown as sticks. Ligands are represented as sticks and colored as follows: yellow for CYM5442, pink for HY-X-1011, light blue for Ponesimod, cyan for SAR247799, red for S1P (from 7VIE), and yellow-orange for Siponimod (from 7EW1). Dashed gray circles highlight differences in binding poses between Siponimod and CYM5442, HY-X-1011, or Ponesimod.

Compared to previously reported structures of S1P bound to S1PR1 and S1PR3, the central regions of CYM5442, HY-X-1011, Ponesimod, and SAR247799 adopt a characteristic “curved” conformation, positioning them closer to two nonconserved residues—L^6.55^ and L^7.39^—located at the TM6 and TM7. Structural superposition of S1PR1 with S1PR3 revealed that these four agonists with a larger molecular width and branched moieties cause steric clashes solely with F263^6.55^, I284^7.39^, or both (Fig 6A–6D). Furthermore, compared to S1P-bound S1PR1 and S1PR3, the branched moieties at the tail portions of CYM5442, HY-X-1011, and Ponesimod extend deeper into the subpocket formed at the TM5-TM6 interface of S1PR1. This subpocket is composed of F210^5.47^, T207^5.44^, F273^6.52^, and L276^6.55^ in S1PR1, corresponding to F204^5.47^, I201^5.44^, F260^6.52^, and F263^6.55^ in S1PR3 and F201^5.47^, V198^5.44^, F268^6.52^, and L271^6.55^ in S1PR5 (Fig 6E–6L). However, compared to F276^6.55^ in the active state of S1PR1, F263^6.55^ in S1P-bound S1PR3 adopts a different conformation, significantly reducing the volume of this subpocket and causing steric hindrance with the branched moieties at the tail portions of CYM5442, HY-X-1011, and Ponesimod upon structural alignment of S1PR1 with S1PR3 (Fig 6E–6H). This likely diminishes the binding affinity of these three agonists toward S1PR3. Similarly, when aligning S1PR1 with S1PR5, compared to Siponimod bound in S1PR5, the branched moieties at the tail portions of CYM5442, HY-X-1011, and Ponesimod insert more deeply into the subpocket located at the TM5-TM6 interface of S1PR1. Notably, Siponimod is an approved drug for treating MS with comparable affinity for both S1PR1 and S1PR5 [22,25]. Moreover, the lower branched moieties of these three agonists are close to F210^5.47^, and the nonconserved residue T207^5.44^ in S1PR1 corresponds to V198^5.44^ in S1PR5, potentially influencing receptor activation. These structural observations align well with our BRET assay results. Based on the above analysis, we conclude that the enhanced selectivity of compounds CYM5442, HY-X-1011, Ponesimod, and SAR247799 over S1PR3 and S1PR5 stems from their binding orientation toward TM5-TM7 and branched substituents.

### 2.6 Comparison of diverse agonists

To investigate whether other S1PR1-selective agonist exhibit similar characteristics that could potentially enhance their selectivity for S1PR1, we analyzed the structures of Siponimod (BAF312), CBP307, and SEW2871 in complex with S1PR1 [31,33]. All three agonists bind in close proximity to TM5-TM7, compared to S1P bound in S1PR3 (S11A–S11C Fig).

Siponimod exhibits potent activation of S1PR1 and S1PR5 with minimal activity toward S1PR3 [45], as consistently characterized by our BRET-based Gi dissociation assays [41]. The branched moieties of Siponimod, particularly the phenyl trifluoromethyl group, could cause steric clashes with I284^7.39^, F263^6.55^, and F260^6.52^ in S1PR3, which likely contribute to its reduced potency at this receptor subtype (S11A Fig). Although the trifluoromethyl group of Siponimod inserts into the subpocket between TM5 and TM6 in S1PR1, in S1PR5 this group is positioned closer to TM3 and does not fully penetrate the subpocket between TM5 and TM6. Nevertheless, the polar head of Siponimod engages in interactions with K34, N101^2.60^, R120^3.28^, and E121^3.29^. These residues are fully conserved in S1PR5 and likely contribute to S1PR5 activation by Siponimod, maintaining a comparable potency to that observed for S1PR1.

Additionally, a similar phenomenon was observed for HY-X-1011. The EC_50_ value of HY-X-1011 for S1PR3 is higher than those of CYM5442, Ponesimod, and SAR247799 for the same receptor (Fig 2B, 2E, and S3 Table). The primary distinction between HY-X-1011 and the other three agonists lies in its head group, allowing for more extensive interactions with polar residues located in the top region of the binding pocket. These interactions potentially contribute to the higher potency of HY-X-1011 toward S1PR3.

CBP307 also exhibits enhanced selectivity for S1PR1 over S1PR3 and S1PR5 in previously report [33,41]. Although there are no branched groups near the tail part of CBP307 that could insert into the subpocket between TM5 and TM6, CBP307 still exhibits higher potency toward S1PR1 compared to S1PR5. We attribute this enhanced selectivity primarily to the influence of nonconserved amino acids, particularly the difference between M124 in S1PR1 and V115 in S1PR5 as well as to the interaction between CBP307’s head group and the conserved polar residues in the top region of the binding pocket (S11B Fig). Our previous studies have shown that the M124A mutation in S1PR1 reduces the potency of CBP307, decreasing the EC_50_ by approximately one order of magnitude [33]. Furthermore, the fluorine atom located on the benzene ring near the head group of CBP307 increases the molecular width, which may hinder the faster and more stable binding of CBP307 to S1PR3, thereby reducing its activation potency. Additionally, other nonconserved residues within the binding pockets of S1PR1 and S1PR3 may further contribute to the higher activity and selectivity of CBP307 for S1PR1 over S1PR3.

SEW2871 has been reported as a selective S1PR1 agonist, with no activity observed at S1PR2-S1PR5 even at concentrations up to 10 μM [46]. SEW2871 exhibits a significantly shorter molecular length compared to all above analyzed agonists (S11C Fig). Besides, the introduction of two trifluoromethyl groups significantly increased the molecular width. Trifluoromethyl group attached to the thiophene ring at the distal end of the molecule inserts into the subpocket formed by transmembrane helices TM5 and TM6, positioning the thiophene ring in close proximity to TM6. This trifluoromethyl group experiences steric clashes with F263^6.55^ and F260^6.52^ in S1PR3 (S11C Fig). Additionally, we observe interactions between the trifluoromethyl group located at the molecular head with residues N101^2.60^ and E121^3.29^. Notably, prior studies have indicated that the N101^2.60^ and E121^3.29^ mutation exerts only a weak effect on the activation of S1PR1, suggesting a limited role for these residues in S1PR1 activation [31].

## 3. Discussion

In this study, we integrated structural analysis, molecular dynamics simulations, and pharmacological assays to elucidate the molecular basis for the selectivity of CYM5442, HY-X-1011, Ponesimod, and SAR247799 toward S1PR1. Specifically, selectivity arises from a combination of nonconserved residues in both the ligand-binding pocket and the Gi-protein interaction interface, the distinct binding orientation toward TM5-TM7 and branched substituents increase the agonist’s effective width, which lead to steric clashes with three residues in S1PR3, and the presence of branched moieties on three agonists that restrict deep insertion into the base of the binding pocket, particularly in S1PR3 and S1PR5. Additionally, we analyzed three other S1PR1 selective agonists and found that their activity profiles toward S1PR1, S1PR3, and S1PR5 align with the characteristics summarized above. Besides, reducing polar interactions with conserved polar residues in the top region of the binding pocket may enhance selectivity for S1PR1. Moreover, the relatively broad molecular width of the agonist sterically hinders its binding into S1PR2 and S1PR4 pocket by nonconserved residue pairs bearing bulky side chains. These findings provide a structural framework for the rational development of next-generation highly selective S1PR1 agonists with enhanced therapeutic potential.

Given that the activation mechanisms of different G protein subtypes (Gi/o, Gs, Gq, and G12/13) coupled to S1PR1 have not yet been clearly elucidated, in this study, we specifically compared selective agonists that adopt extended conformations within the orthosteric binding pocket of S1PR1 to activate S1PR1 and its downstream Gi1 signaling. Whether the observed common characteristics in S1PR1-mediated Gi1 activation extend to other G protein subtypes remains to be determined and warrants further investigation.

GPCR signaling via G proteins can be terminated by arrestin binding. Receptor phosphorylation by G protein-coupled receptor kinases (GRKs) promotes arrestin recruitment, which blocks further G protein interaction and initiates receptor internalization. Many S1PR modulators activate both G protein-dependent and arrestin-dependent signaling pathways, which may result in opposing physiological effects. Therefore, developing S1PR modulators with bias toward either G protein or arrestin signaling is crucial for creating more effective and targeted therapeutics targeting S1PR1. SAR247799, a previously reported Gi-biased agonist of S1PR1, exhibits endothelial protective properties and has recently been shown to preserve endothelial barrier integrity in inflammatory bowel disease (IBD) models [37,40]. Additionally, this study also reports the structure of SAR247799 bound to S1PR1. In our study, we analyzed SAR247799 recognition and activation mechanisms of S1PR1 and initiating downstream Gi signaling. However, the molecular basis for its G protein bias remains unclear. Structural determination of the S1PR1-β-arrestin complex could provide insights into the mechanisms underlying G protein or β-arrestin bias, thereby facilitating the rational design of biased S1PR1-selective agonists with improved therapeutic profiles.

Due to differences in expression patterns and downstream signaling pathways among S1PR1-S1PR5, the development of highly selective agonists targeting individual subtypes, such as S1PR2-S1PR5, holds promise for the targeted treatment of specific diseases. A previous study reported ONO-5430608 as an inverse agonist of S1PR5 [35]. Notably, unlike typical S1PR1 agonists, ONO-5430608 adopts a different conformation when bound within the S1PR5 ligand-binding pocket, and its selectivity for S1PR5 appears to be influenced by the conformation of residue Y89. However, further research is still needed to elucidate the structural basis and pharmacological potential of selective small-molecule agonists for S1PR2-S1PR5. Additionally, the identification of nonconserved allosteric sites across S1PR1-S1PR5 may offer an additional strategy for developing S1PRs-subtype-selective agonists with enhanced therapeutic properties [47].

### Methods section

#### ScFv16 Purification.

ScFv16 was subcloned into the pFastBac vector and expressed in *Trichoplusia ni* Hi5 insect cells using the Bac-to-Bac system, exactly following the established protocol [33]. Briefly, Hi5 cells were infected by ScFv16 baculovirus (amplified in Sf9 cells) for 96 hours. The medium was collected by centrifugation, pH-balanced to 8.0, and then supplemented with 1 mM NiCl₂ and 5 mM CaCl₂. After incubating for 1 hour at RT, the supernatant was mixed with Ni-Sepharose resin (GE Healthcare) for another 1 hour. The resin was then collected (800 × *g*, 10 min, 4 °C) and transferred to a gravity column. The column was washed with wash buffer C (20 mM HEPES pH 7.5, 100 mM NaCl, 20 mM imidazole) and eluted with a high-imidazole buffer (wash buffer C containing 250 mM imidazole). HRV-3C protease was added to remove the C-terminal His-tag. Following the tag removal, size-exclusion chromatography was performed on a Superdex 200 Increase 10/300 GL column (GE Healthcare) pre-equilibrated with 20 mM HEPES (pH 7.5) and 100 mM NaCl. The target fractions were concentrated, flash-frozen in liquid nitrogen, and stored at −80 °C.

#### S1PR1 and G protein expression and complex purification.

As previously described [33], the full-length WT human S1P1 with the BRIL protein fused to its N-terminus was subcloned into the pFastBac vector. Meanwhile, the WT Gαi1 and Gβ1γ2 were respectively inserted into the pFastBac vector and pFastBac-Dual vector. The complex was co-expressed in *Spodoptera frugiperda* (Sf9) insect cells with baculoviruses encoding S1P1, WT Gαi1, and WT Gβ1γ2 at a 1:1:1 multiplicity of infection. Infected cells were harvested after 48 hours, followed by flash-freezing in liquid nitrogen, and stored at −80 °C until use. To stabilize the complex, all purification steps were performed in the presence of 10 μM ligands (CYM5422, HY-X-1011, Ponesimod, SAR247799). The cell pellets were thawed and resuspended in lysis buffer (25 mM HEPES pH 7.5, 150 mM NaCl, 5% glycerol, 10 mM MgCl₂, 20 mM KCl, 5 mM CaCl₂, 1 mM MnCl₂, 100 μM PMSF, 2 μg/mL aprotinin, 2 μg/mL pepstatin) and sequentially treated with 25 mU/mL apyrase (1 hour, room temperature) and 1% DDM/0.1% CHS (2 hours, 4 °C). After centrifugation (39,191 × *g*, 30 min, 4 °C), the supernatant was incubated with anti-Flag resin (GenScript) in wash buffer A (25 mM HEPES pH 7.5, 150 mM NaCl, 5% glycerol, 5 mM MgCl₂, 5 mM CaCl₂, 0.01% LMNG, 0.001% CHS). Bound proteins were eluted using wash buffer A supplemented with 200 μg/mL Flag peptide. The eluate was further purified by Ni-NTA affinity chromatography. After loading onto Ni-NTA resin (Qiagen), the column was washed with wash buffer B (25 mM HEPES pH 7.5, 150 mM NaCl, 5 mM MgCl₂, 25 mM imidazole, 0.01% LMNG, 0.001% CHS) and eluted with a high-imidazole buffer (wash buffer B containing 250 mM imidazole). The eluted complex was concentrated to less than 2 mL using an Amicon Ultra Centrifugal Filter (MWCO 100 kDa) and incubated with excess scFv16 for 2 hours on ice. Final purification was achieved by size-exclusion chromatography (Superdex 200 Increase 10/300 GL, GE Healthcare) in SEC buffer (25 mM HEPES pH 7.5, 150 mM NaCl, 0.00075% LMNG, 0.00025% GDN, 0.000075% CHS, 100 μM TCEP). Monomeric fractions containing the complex were concentrated to 10–15 mg/mL for cryo-EM grid preparation.

#### Molecular cloning of constructs used in BRET assay.

The full-length WT human S1PR1, S1PR3, S1PR5, and mutants were cloned into a pcDNA3.1 vector with a HA signal peptide, an N-terminal Flag tag. The construct was generated with a standard PCR-based strategy and homologous recombination (1.1 × S4 Fidelity PCR Mix, Beijing Genesand Biotech Co.).

#### Cell culture of BRET assay.

HEK293T cells (ATCC CRL-11268; mycoplasma free) were maintained, passaged, and transfected in DMEM medium containing 10% FBS, 100 U/ml penicillin, and 100 μg/ml streptomycin (Gibco-ThermoFisher) in a humidified atmosphere at 37 °C and 5% CO_2_. After transfection, cells were plated in DMEM containing 2% dialyzed FBS for BRET assay.

#### Bioluminescence resonance energy transfer assay (BRET).

BRET assays were used to measure agonists induced S1PR1/3/5 (WT or mutants) activation coupled Gi signal [48]. HEK293T cells (ATCC CRL-11268; mycoplasma free) were co-transfected in a 1:1:1:1 ratio of receptor: Gαi1-Rluc8: Gβ1: Gγ2-GFP_2_ with polyethyleneimine. After at least 18 hours, transfected cells were harvested and reseeded in opaque white bottom 96-well assay plates (Beyotime) at a density of 30,000–50,000 cells per well in media (DMEM added 2% dFBS). The next day, the medium was decanted. Cells were incubated in 40 μL 7.5 μM coelenterazine 400a (Goldbio) in drug buffer (1 × Hank’s balanced salt solution (HBSS), 20 mM HEPES, pH 7.4, 0.1% BSA) for 2 min, and then treated with 20 μL compounds (CYM5442, HY-X-1011, Ponesimod, and SAR247799) prepared in drug buffer at serial concentration gradient for an additional 5 min. Plates were read in an LB940 Mithras plate reader (Berthold Technologies) with 395-nm and 510-nm emission filters with 1 s per well integration times. BRET ratios were calculated as the ratio of GFP2 emission (510 nm) to Rluc8 emission (395 nm) and analyzed in GraphPad prism 9.0 or GraphPad Prism 10. Ponesimod, CYM5442, and SAR247799 were purchased from MedChemExpress (MCE). HY-X-1011 was synthesized by MCE or by collaborators. Data were normalized to 100% of maximal response of WT stimulation and analyzed using nonlinear regression curve fit log (agonist vs. response). The mean values and SEM/SD values are generated from all repeated samples. Mean values and SEM/SD were automatically calculated for all replicates in GraphPad.

#### Cryo-EM sample preparation, data acquisition, and data processing.

The purified complex was applied to glow-discharged 300-mesh alloy grids (Quantifoil 300 mesh, Au R1.2/1.3 and M024-Au300-R1.2/1.3) and subsequently vitrified using Vitrobot Mark IV. The images were collected in the counted-Nanoprobe mode on a 300 kV Titan Krios Gi3 electron microscope (Thermo Fisher Scientific) equipped with Gatan K3 Summit detector and GIF Quantum energy filter (slit width 20 eV). All movie stacks with 50 frames were collected using SerialEM software at a nominal magnification of 105,000×, a pixel size of 0.85 Å, and a defocus range of −1.2 μm to −1.8 μm. Each movie stack for S1PR1-Gi1-CYM5442, S1PR1-Gi1-HY-X-1011, S1PR1-Gi1-Ponesimod, and S1PR1-Gi1-SAR247799 was recorded for 2.8 s, 2.2 s, 2.3 s, and 2.2 s, respectively, corresponding to a total dose of 53.83 e^−^/Å^2^, 51.73 e^−^/Å^2^, 52.56 e^−^/Å^2^, and 55.63 e^−^/Å^2^, respectively. 3303 movies, 3159 movies, 3141 movies, and 2906 movies were collected for S1PR1-Gi1-CYM5442, S1PR1-Gi1-HY-X-1011, S1PR1-Gi1-Ponesimod, and S1PR1-Gi1-SAR247799, respectively.

For S1PR1-Gi1-CYM5442, S1PR1-Gi1-HY-X-1011, and S1PR1-Gi1-Ponesimod data, Data processing was performed using cryoSPARC [49]. Movies frames were aligned using Patch motion. CTF estimation was performed using Patch CTF. Particles were first picked using a blob picker. Particle picking of all micrographs was performed by a template picker. 2,204,113, 3,670,342, and 3,401,413 particles were extracted. Afterwards, particles sets were selected and refined by iterative 2D classification, ab initio reconstruction, and heterogeneous refinement. Finally, 198,838, 1,019,239, and 529,510 particles were selected for S1PR1-Gi1-CYM5442, S1PR1-Gi1-HY-X-1011, and S1PR1-Gi1-Ponesimod data, respectively. After one round of ab initio reconstruction, non-uniform and local refinement, 3.69 Å, 2.79 Å, and 2.79 Å map were refined out for S1PR1-Gi1-CYM5442, S1PR1-Gi1-HY-X-1011, and S1PR1-Gi1-Ponesimod data, respectively.

For S1PR1-Gi1-SAR247799 data, movies were imported into RELION 5.0 [50]. Beam-induced motion was corrected using MotionCor2 [51], after which the micrographs were imported into CryoSPARC for contrast transfer function (CTF) parameter estimation using PatchCTF. 4,653,961 particles were picked and extracted in a pixel size of 1.7 Å. After 2D classification, 1,364,671 particles were selected for ab-initio reconstruction and heterogeneous refinement. The retained 359,408 particles were re-extracted in RELION in a pixel size of 0.85. These particles underwent additional sieving using CryoSIEVE [52], resulting in a final selection of 94,216 particles. The final CTF refinement, particle polishing, non-uniform refinement and local refinement result in density map of 3.0 Å resolution.

#### Model building and refinement.

The initial complex model was built using the structure of S1PR1-Gi-S1P (PDB code: 7VIE) as templates. The structure model of S1PR1-Gi-S1P is fitted into the density map and manually adjusted and fixed in COOT [53]. The restraint files of CYM5442, HY-X-1011, Ponesimod, and SAR247799 were generated by Phenix. elbow package [54]. The complete model was finally refined in Phenix using real-space refinement with secondary structure and geometry restraints [55] and was checked in COOT. Overfitting of the model was checked by refining the model using one of the two independent maps from gold-standard refinement and calculating FSC against both half maps [56]. The final model was validated using Molprobity [57] (S1 Table). Structural figures were prepared in PyMOL (https://pymol.org/2/), UCSF Chimera [58], and UCSF ChimeraX [59].

#### Molecular dynamics simulation.

Four agonists were docked into previously reported inactive structure (S1PR1 PDB ID: 3V2Y) and active structures (S1PR1 PDB ID: 7VIE, S1PR3 PDB ID: 7EW3, and S1PR5 PDB ID: 7EW1). The inactive structure model of S1PR3 and S1PR5 was homology modeled by modeler using the inactive S1PR1 structure (PDB ID: 3V2Y) as the template [60]. Agonists docked inactive structures of receptors were used as the start inactive state. Agonists docked active structures of receptors were used as the end active state. AutoDock 4.2 [61] was used to dock the ligands into the binding pocket of the apo inactive/active receptors. In all MD simulations, only receptors and ligands were used, with G protein complex and other protein removed from the structures. The Membrane Builder module in CHARMM-GUI server [62] was used to prepare the simulation inputs, a membrane of pre-equilibrated (310 K) POPC lipids based on the OPM database [63] alignment, TIP3P solvent with 0.15 M Na^+^/Cl^−^ ions and the CHARMM36 force field [64]. The force field of the ligands was generated by the CGenFF program [65]. All MD simulations were performed using GROMACS-2019.4 [66]. The CHARMM36 force field was used to describe the interactions in the system. Energy minimization was performed for 10,000 steps by the steepest descent algorithm and then by the conjugate gradient algorithm. Then a 100 ps NVT simulation was performed at 310 K for solvent equilibration, followed by a 1 ns NPT equilibration to 1 atm using the Berendsen barostat [67]. All MD production simulations were performed with a time-step of 1 fs and a length of 100 ns using Parrinello-Rahman barostat [68]. In all constant temperature simulations, the Bussi (velocity-rescaling) thermostat was used [69]. Long-range electrostatic interactions were treated by the particle-mesh Ewald method [70]. The short-range electrostatic and van der Waals interactions both used a cutoff of 10 Å. All H-bonds were constrained by the LINCS algorithm [71].

#### Path searching.

Targeted MD simulations (tMD) were carried out using GROMACS-2019.4 and PLUMED-2.5.3 [72], pulling the S1PR1/3/5-ligands complexes from the inactive states to the active states. The inactive structures were relaxed by a 100-ns MD simulation, and the active structures were energy-minimized, followed by a 100-ps NVT and a 1-ns NPT equilibration. The additional bias potential introduced in tMD takes a simple harmonic form $U(s)={\frac{k}{\text{2}}(s-s_{\text{0}})}^{\text{2}}$, where k is the spring force constant, $s_{\text{0}}$ represents the target structure. k was set to 1000 kJ/(mol Å^2^), biasing on heavy atoms of the nonloop parts of receptors and the ligands, while structure alignments were performed using C-alpha atoms of the non-loop parts of receptors.

To find the MFEPs, which are more physically natural than the initial paths, the TAPS method [42] was used to do path optimization. The TAPS simulation was performed using an in-house python script incorporating GROMACS, PLUMED, and Concorde [73]. The simulations were performed in the NVT ensemble at 310 K, using the velocity-rescale thermostat [69]. The initial path was obtained by selecting conformations with a gap of 1.0 Å from the tMD trajectory. The RMSD between the conformations was computed using all heavy atoms of the nonloop parts of receptors and ligands, while structure alignments were performed using C-alpha atoms of the nonloop parts of receptors. In each TAPS iteration, 1000 ps sampling was performed in total. Gaussians of height 0.25 kJ/mol and width 0.5 were deposited every 0.01 ps, with frames recorded at the same frequency. After the optimization, convergence was evaluated by Multidimensional Scaling (MDS) method [74] and PCV-z analysis [75].

#### Free energy calculation.

To evaluate the probability of receptor activation, umbrella sampling [76] was used to do the free energy calculation. Umbrella sampling was performed using GROMACS-2019.4 and PLUMED-2.5.3. The free energy profiles of the MFEPs were calculated along PCV-s, which represents the progress along the MFEP. The sampling in each window was restrained within 1.5 Å of MFEP through a harmonic wall potential with a force constant of 20,000 kJ/(mol•Å^4^) at PCV-z = 2.25 Å^2^. The RMSD among the conformations, which was computed by all heavy atoms of the nonloop parts of receptors and ligands, was chosen as the CV. Structure alignment was performed using C-alpha atoms of the nonloop parts of receptors. The window size was chosen as 0.5 Å along the MFEP. For each window, a force constant of 200 kJ/mol was employed. Each window was simulated for at least 10 ns. A complete free energy profile was evaluated with the weighted histogram analysis method (WHAM) [77]. When the iteration process of WHAM reached convergence, the mean value and standard statistical error (error bar) of the free energy were calculated in each window from the last 10 WHAM iterations.

## Supporting information

S1 Fig Agonists exhibit distinct activation profiles across S1PR subtypes. (**A–D**) Two-dimensional structure of four agonist molecules: CYM5442, HY-X-1011, Ponesimod, and SAR247799. (**E**) BRET-based Gi1 dissociation assay measuring S1P-induced activation of S1PR1, S1PR3, and S1PR5. (**F**) BRET-based Gi1 dissociation assay assessing activation of S1PR2 by CYM5442, HY-X-1011, Ponesimod, SAR247799, and S1P. (**G**) BRET-based Gi1 dissociation assay assessing activation of S1PR4 by CYM5442, HY-X-1011, Ponesimod, SAR247799, and S1P. Data are presented as mean ± SD; *n* = 3 in panels (E–G). The underlying data for S1E–S1G Fig can be found in S6 Data.
(TIF)

S2 Fig Purification of the S1PR1-Gi1 complex bound with CYM5442, HY-X-1011, Ponesimod, and SAR247799. (**A–D**) Gel filtration chromatography elution profile of the four complexes and SDS-PAGE of the eluted complex.
(TIF)

S3 Fig Structural determination of the S1PR1-Gi1 complex bound with CYM5442, HY-X-1011, Ponesimod, and SAR247799. (**A–D**) Flowchart for processing cryo-EM data of S1PR1-Gi1 bound with CYM5442, HY-X-1011, Ponesimod, or SAR247799. Representative micrographs, 2D classes, local resolution of the final map estimated via cryoSPARC, and Fourier shell correlation (FSC) curves of the final refined cryo-EM map are displayed. (**E**) FSC between the map and model of the four complexes. FSC curve of the final refined model against the full map. (**F–I**) Density maps of the transmembrane helix of S1PR1 in the four complexes are shown as meshes.
(TIF)

S4 Fig Densities of key residues and agonists within the S1PR1 binding pocket. Residues and agonist are shown in sticks. Residue densities and agonist densities are shown in mesh in panels (**A–D**).
(TIF)

S5 Fig Conformational transitions of S1PR1 upon agonist binding. (**A–D**) The transmembrane (TM) helix shifts between the active form of S1PR1 (marine-colored) bound with CYM5442, HY-X-1011, Ponesimod, or SAR247799 and the inactive form of the S1PR1-ML056 complex (gray; PDB: 3V2Y). Red arrows indicate notable shifts in TM6, with corresponding distance measurements. (**E–H**) Conformational changes in key residues at the bottom of the pockets between the active form S1PR1 bound with CYM5442, HY-X-1011, Ponesimod, or SAR247799 and the inactive form S1PR1. Residues and molecules are shown as sticks and colored yellow (CYM5442), pink (HY-X-1011), light blue (Ponesimod), cyan (SAR247799), and gray (ML056). (**I–T**) Conformational changes in the E^3.49^R^3.50^Y^3.51^ motif, P^5.50^I^3.40^F^6.44^ motif, and N^7.49^P^7.50^xxY^7.53^ motif between the active form of S1PR1 bound with CYM5442, HY-X-1011, Ponesimod, or SAR247799 and the inactive form of S1PR1.
(TIF)

S6 Fig Extended mutational analysis of nonconserved residues in S1PR1/S1PR3 selectivity. (**A–F**) BRET-based Gi1 dissociation assays showing activation responses of S1PR1 and S1PR3 swapped mutants (S1PR1_S129G_, S1PR3_G123S_, S1PR1_T207I_, and S1PR3_I201T_) induced by Ponesimod or SAR247799. Data are presented as mean ± SD; *n* = 3. The underlying data for S6A–S6F Fig can be found in S7 Data.
(TIF)

S7 Fig Extended mutational analysis of nonconserved residues in S1PR1/S1PR5 selectivity. (**A–H**) BRET Gi1 dissociation assay of S1PR1 and S1PR5 swapped mutants (S1PR1_M124V_, S1PR5_V115M_, S1PR1_S129T_, and S1PR5_T120S_) activation responses induced by CYM5442, HY-X-1011, Ponesimod, or SAR247799. The data are presented as the means ± SD; *n* = 3. The underlying data for S7A–S7H Fig can be found in S8 Data.
(TIF)

S8 Fig Agonist-induced rearrangements among residues V209^5.46^, F133^3.41^, and L213^5.50^. (**A**) Superimposition of active S1PR1 structures bound to CYM5442, HY-X-1011, Ponesimod, or SAR247799 with the inactive form of S1PR1 (PDB: 3V2Y). Residues are shown as sticks, and red lines indicate distance differences between F133^3.41^, and L213^5.50^ in the active and inactive structures. (**B–E**) Densities of V209^5.46^, F133^3.41^, and L213^5.50^ in four agonists bound structures are shown in mesh.
(TIF)

S9 Fig Nonconserved residues at the S1PR1/S1PR5-Gi1 binding interface influence receptor activation. (**A**) Superimposition of the Gi-binding surface of S1PR1-Gi bound with CYM5442, S1PR3 (PDB: 7EW3), and S1PR5 (PDB: 7EW1). Nonconserved residues of S1PR1 (marine), S1PR3 (gray), and S1PR5 (yellow-orange) and D350 of Gi1 are shown as sticks. Gi1 is colored in green. (**B–E**) BRET-Gi1 dissociation assay showing activation responses of S1PR1 and S1PR5 swapped mutants (S1PR1_R78A_ and S1PR5_A69R_) induced by CYM5442, HY-X-1011, Ponesimod, or SAR247799. The data are presented as the means ± SD; *n* = 3. The underlying data for S9B–S9E Fig can be found in S9 Data.
(TIF)

S10 Fig Nonconserved residues obstruct four agonists binding with S1PR2 and S1PR4. (**A–D**) Superimposition of the structures of CYM5442, HY-X-1011, Ponesimod, or SAR247799 bound S1PR1 with S1P bound S1PR2 (PDB: 7T6B). A112^3.32^, F274^7.39^ and H271^7.31^ are nonconserved in S1PR1, whereas N89^2.60^, R108^3.28^, and E109^3.29^ are conserved. Red circles highlight steric clashes between the agonist and residues of S1PR2. (**E–H**) Superimposition of the structures of CYM5442, HY-X-1011, Ponesimod, or SAR247799 bound S1PR1 with S1PR4 (predict model by SWISS-MODEL). L125^3.32^ and M289^7.35^ are nonconserved in S1PR1. N102^2.60^ and E122^3.29^ are conserved in S1PR1. A gay dashed rectangle and a gray arrow indicate that the bulky tail of the agonists would clash with E122^3.29^ and M289^7.35^ when binding to the S1PR4 ligand-binding pocket, due to the restricted distance between these two residues. Yellow dashed lines represent distances between the agonists and specific residues, with values labeled accordingly in panel (A–H).
(TIF)

S11 Fig Structural analysis reveals consistent binding features among S1PR1-selective agonists. (**A**) Superimposition the structures of S1PR1 bound with Siponimod (PDB: 7EO4), S1PR3 bound with S1P (PDB: 7EW3), and S1PR5 bound with Siponimod (PDB: 7EW1). (**B**) Superimposition the structure of S1PR1 bound with CBP307 (PDB: 7VIG), S1PR3 bound with S1P (PDB: 7EW3), and S1PR5 (PDB: 7EW1). A red dashed rectangle indicates the nonconserved residues: V115 in S1PR5 and M124 in S1PR1. (**C**) Superimposition the structures of S1PR1 bound with SEW2871 (PDB: 7EW7), S1PR3 (PDB: 7EW3), and S1PR5 bound with Siponimod (PDB: 7EW1). Dashed lines in panels (A–C) indicate hydrogen bonds. Red circles highlight steric clashes between the agonist and residues of S1PR3.
(TIF)

S1 Table Cryo-EM data collection, refinement, and validation statistics. (DOCX)

S2 Table Activation parameters of S1PR1 mutants across four agonists measured by BRET assay. a, b, and c represent data from different batches and wild-type data in the same batches were used for normalization. EC_50_ and Emax values represents average from three independent experiments performed in duplicate. pEC_50_ is presented by mean ± SEM. N.D., indicates that no equilibrium response was achieved at maximum agonist concentration for a reliable curve fitting. “-” denotes complete loss of activation.
(DOCX)

S3 Table Activation parameters of S1PRs swapped mutants tested by BRET assay. a, b, c, and d represent data from different batches and wild-type data of each receptor in the same batches were used for normalization. EC_50_ and Emax values represents average from three independent experiments performed in duplicate. pEC_50_ is presented by mean ± SEM. “-” denotes complete loss of activation.
(DOCX)

S1 Data Underlying data for Fig 1A–1H. (XLSX)

S2 Data Underlying data for Fig 2E and S2 Table. (XLSX)

S3 Data Underlying data for Fig 3E–3L. (XLSX)

S4 Data Underlying data for Fig 4E–4L. (XLSX)

S5 Data Underlying data for Fig 5E–5G. (XLSX)

S6 Data Underlying data for S1E–S1G Fig. (XLSX)

S7 Data Underlying data for S6A–S6F Fig. (XLSX)

S8 Data Underlying data for S7A–S7H Fig. (XLSX)

S9 Data Underlying data for S9B–S9E Fig. (XLSX)

S1 Raw Image Uncropped Coomassie blue-stained SDS-PAGE gel used for S2A–S2D Fig. (TIF)

## Abbreviations

BRET: bioluminescence resonance energy transfer
CNS: central nervous system
cryo-EM: cryo-electron microscopy
CTF: contrast transfer function
eNOS: endothelial nitric oxide synthase
GRKs: G protein-coupled receptor kinases
HBSS: Hank’s balanced salt solution
IBD: inflammatory bowel disease
MCE: MedChemExpress
MDS: Multidimensional Scaling
MFEP: minimum free energy path
MS: multiple sclerosis
NO: nitric oxide
S1P: Sphingosine-1-phosphate
S1PRs: sphingosine-1-phosphate receptors
TAPS: traveling-salesman based automated path searching
tMD: Targeted MD simulations
TM: transmembrane helices
TM6: transmembrane helix 6
VECs: vascular endothelial cells
WHAM: weighted histogram analysis method
WT: wild-type.
