# Supplementary material for: Structural insights into subtype-specific agonist recognition by sphingosine-1-phosphate receptors
Source: PLoS Biol. 2026 Apr 10;24(4):e3003381. doi: 10.1371/journal.pbio.3003381 (PMC13089896; doi:10.1371/journal.pbio.3003381)
Supplement: S1 Table — (DOCX) [file pbio.3003381.s012.docx]

**S1 Table. Cryo-EM data collection, refinement and validation statistics**

|  | S1PR1- CYM5442  (EMDB-65218)  (PDB 9VNY) | S1PR1-HY-X-1011  (EMDB-65219)  (PDB 9VNZ ) | S1PR1-Ponesimod  (EMDB-65220)  (PDB 9VO0) | S1PR1-SAR247799  (EMDB-65221)  (PDB 9VO1) |
| --- | --- | --- | --- | --- |
| **Data collection and processing** |  |  |  |  |
| Magnification | 105,000 | 105,000 | 105,000 | 105,000 |
| Voltage (kV) | 300 | 300 | 300 | 300 |
| Electron exposure (e–/Å^2^) | 53.83 | 51.73 | 52.56 | 55.63 |
| Defocus range (μm) | -1.2- -1.8 | -1.2- -1.8 | -1.2- -1.8 | -1.2- -1.8 |
| Pixel size (Å) | 0.85 | 0.85 | 0.85 | 0.85 |
| Symmetry imposed | C1 | C1 | C1 | C1 |
| Initial particle images (no.) | 2,204,113 | 3,670,342 | 3,401,413 | 4,653,961 |
| Final particle images (no.) | 198,838 | 1,019,239 | 529,510 | 94,216 |
| Map resolution (Å)  FSC threshold | 3.69  0.143 | 2.79  0.143 | 2.79  0.143 | 3.0  0.143 |
| Map resolution range (Å) | 2.8-4.4 | 2.6-4.2 | 2.6-4.2 | 2.8-4.5 |
|  |  |  |  |  |
| **Refinement** |  |  |  |  |
| Initial model used (PDB code) | 7VIE | 7VIE | 7VIE | 7VIE |
| Model resolution (Å)  FSC threshold | 3.7/3.9  0.143/0.5 | 2.8/3.0  0.143/0.5 | 2.8/3.0  0.143/0.5 | 2.8/3.0  0.143/0.5 |
| Model composition  Non-hydrogen atoms  Protein residues  Ligands | 8873  1137  1 | 8890  1135  1 | 8906  1136  1 | 8861  1134  1 |
| *B* factors (Å^2^)  Protein  Ligand | 98.33  124.24 | 78.05  150.33 | 111.66  20.00 | 135.43  181.76 |
| R.m.s. deviations  Bond lengths (Å)  Bond angles (°) | 0.004  0.841 | 0.005  0.672 | 0.004  0.678 | 0.003  0.597 |
| Validation  MolProbity score  Clashscore  Poor rotamers (%) | 1.97  13.20  0.00 | 1.50  6.62  0.00 | 1.85  9.92  0.00 | 1.81  7.60  0.10 |
| Ramachandran plot  Favored (%)  Allowed (%)  Disallowed (%) | 95.08  4.56  0.36 | 97.31  2.69  0.00 | 95.25  4.48  0.27 | 94.25  5.75  0 |
