# Supplementary material for: Structural insights into subtype-specific agonist recognition by sphingosine-1-phosphate receptors
Source: PLoS Biol. 2026 Apr 10;24(4):e3003381. doi: 10.1371/journal.pbio.3003381 (PMC13089896; doi:10.1371/journal.pbio.3003381)
Supplement: S2 Table — a, b, and c represent data from different batches and wild-type data in the same batches were used for normalization. EC50 and Emax values represents average from three independent experiments performed in duplicate. pEC50 is presented by mean ± SEM. N.D., indicates that no equilibrium response was achieved at maximum agonist concentration for a reliable curve fitting. “-” denotes complete loss of activation. (DOCX) [file pbio.3003381.s013.docx]

**S2 Table. Activation parameters of S1PR1 mutants across four agonists measured by BRET assay**

| CYM5442  EC_50_ (nM) | | | HY-X-1011  EC_50_ (nM) | | | | | Ponesimod  EC_50_ (nM) | | | SAR247799  EC_50_ (nM) | | |
| --- | --- | --- | --- | --- | --- | --- | --- | --- | --- | --- | --- | --- | --- |
| Mutants | pEC_50_±SEM | Emax (WT%) | Mutants | pEC_50_±SEM | | | Emax(WT %) | Mutants | pEC_50_±SEM | Emax(WT %) | Mutants | pEC_50_±SEM | Emax(WT %) |
| S1PR1_WT_^a^ | 26.70  7.57±0.06 | 100.00 | S1PR1_WT_^a^ | | 7.33  8.14±0.07 | 100.00 | | S1PR1_WT_^a^ | 38.09  7.42±0.06 | 100.00 | S1PR1_WT_^a^ | 5.20  8.28±0.03 | 100.00 |
| R120A^a^ | 227.70  6.64±0.07 | 72.65 | N101A^a^ | | 100.20  7.00±0.18 | 42.38 | | N101A^a^ | 87.95  7.06±0.12 | 62.47 | N101A^a^ | 81.99  7.09±0.50 | 11.11 |
| E121A^a^ | 348.00  6.46±0.07 | 100.70 | M124A^a^ | | N.D. | N.D. | | E121A^a^ | 369.60  6.43±0.28 | 30.37 | T109A^a^ | 689.50  6.16±0.21 | 65.26 |
| T207A^a^ | 352.90  6.45±0.08 | 66.23 | F125A^a^ | | N.D. | N.D. | | S129A^a^ | 68.83  7.16±0.33 | 19.93 | E121A^a^ | 72.83  7.14±0.71 | 10.99 |
| S1PR1_WT_^b^ | 14.78  7.83±0.10 | 100.00 | L128A^a^ | | 824.70  6.08±0.09 | 97.37 | | W269^a^ | 1281.00  5.89±0.05 | 108.2 | F125A^a^ | 1172.00  5.93±0.35 | 20.58 |
| N101A^b^ | 1170  5.93±0.19 | 72.08 | S129A^a^ | | 371.10  6.43±0.15 | 58.80 | | L195A^a^ | 246.50  6.61±0.07 | 99.54 | L128A^a^ | 8.42  8.07±0.32 | 23.76 |
| F125A^b^ | 3882.00  5.41±0.55 | 66.97 | F210A^a^ | | 69.59  7.16±0.18 | 48.67 | | T207A^a^ | 187.40  6.73±0.04 | 137.8 | L195A^a^ | - | - |
| L128A^b^ | 1675.00  5.78±0.18 | 112.30 | L276A^a^ | | 6956.00  5.16±0.80 | 59.61 | | F125A^a^ | 641.60  6.19±0.10 | 69.99 | F210A^a^ | 89.68  7.05±0.09 | 100.1 |
| V194A^b^ | 39.17  7.41±0.13 | 93.07 | E294A^a^ | | 1193.00  5.92±0.11 | 121.5 | | L128A^a^ | 310.90  6.51±0.09 | 71.81 | L272A^a^ | - | - |
| F210A^b^ | N.D. | N.D. | L297A^a^ | | 1181.00  5.93±0.23 | 44.15 | | S1PR1_WT_^b^ | 30.27  7.52±0.12 | 100 | L276A^a^ | 1164.00  5.93±0.21 | 39.27 |
| W269A^b^ | 1121.00  5.95±0.13 | 102.70 | S1PR1_WT_^b^ | | 4.45  8.35±0.07 | 100.00 | | F210A^b^ | 1466.00  5.83±0.85 | 33.21 | S1PR1_WT_^b^ | 8.40  8.08±0.06 | 100.00 |
| L272A^b^ | 4456.00  5.35±0.35 | 77.10 | Y29A^b^ | | - | - | | F265A^b^ | 1384.00  5.86±0.08 | 230.8 | R120A | 160.20  6.80±0.24 | 23.63 |
| L276A^b^ | 1425.00  5.85±0.17 | 121.6 | E121A^b^ | | 391.4  6.41±0.15 | 145.0 | | V194A^b^ | 5005.00  5.30±0.18 | 173.9 | S129A^b^ | - | - |
| E294A^b^ | 297.70  6.53±0.24 | 62.31 | L272A^b^ | | N.D. | N.D. | | L272A^b^ | 25.09  7.60±0.15 | 100.4 | L297A^b^ | 560.80  6.25±0.07 | 154.8 |
| L297A^b^ | 1387.00  5.86±0.38 | 58.34 |  | |  |  | | L276A^b^ | 735.10  6.13±0.17 | 70.16 | W269A^b^ | 382.30  6.42±0.24 | 54.91 |
| S1PR1_WT_^c^ | 26.70  7.57±0.06 | 100.00 |  | |  |  | | L297A^b^ | 1742.00  5.76±0.19 | 97.57 |  |  |  |
| M124A^c^ | - | - |  | |  |  | | E294A^b^ | 2154.00  5.67±0.12 | 175.9 |  |  |  |
| S129A^c^ | 1633.00  5.79±0.27 | 46.27 |  | |  |  | |  |  |  |  |  |  |
