# Supplementary material for: Structural insights into subtype-specific agonist recognition by sphingosine-1-phosphate receptors
Source: PLoS Biol. 2026 Apr 10;24(4):e3003381. doi: 10.1371/journal.pbio.3003381 (PMC13089896; doi:10.1371/journal.pbio.3003381)
Supplement: S3 Table — a, b, c, and d represent data from different batches and wild-type data of each receptor in the same batches were used for normalization. EC50 and Emax values represents average from three independent experiments performed in duplicate. pEC50 is presented by mean ± SEM. “-” denotes complete loss of activation. (DOCX) [file pbio.3003381.s014.docx]

**S3 Table. Activation parameters of S1PRs swapped mutants tested by BRET assay**

| CYM5442 | | | | HY-X-1011 | | | Ponesimod | | | SAR247799 | | |
| --- | --- | --- | --- | --- | --- | --- | --- | --- | --- | --- | --- | --- |
| Mutants | EC_50_(nM)  pEC_50_±SEM | | Emax(WT%) | Mutants | EC_50_(nM)  pEC_50_±SEM | Emax(%) | Mutants | EC_50_(nM)  pEC_50_±SEM | Emax(%) | Mutants | EC_50_(nM)  pEC_50_±SEM | Emax(%) |
| S1PR1^a^ | | 12.62  7.90±0.03 | 100.00 | S1PR1^a^ | 9.05  8.04±0.03 | 100.00 | S1PR1^a^ | 34.50  7.46±0.06 | 100.00 | S1PR1^a^ | 8.39  8.08±0.06 | 100.00 |
| S1PR1^a^-L276F/  L297I | | 1422.00  5.85±0.19 | 17.92 | S1PR1^a^-L276F/L297I | 2564.00  5.60±  0.14 | 43.44 | S1PR1^a^-L276F/L297I | 1769.00  5.75±0.40 | 67.55 | S1PR1^a^-L276F/L297I | 383.30  6.42±0.22 | 54.93 |
| S1PR3^a^ | | 1053.00  5.98±0.19 | 100.00 | S1PR3^a^ | 249.60  6.60±0.05 | 100.00 | S1PR1^b^ | 22.15  7.66±0.03 | 100.00 | S1PR1^b^ | 6.16  8.21±0.07 | 100.00 |
| S1PR3^a^-F263L/  I284L | | 40.68  7.39±0.05 | 156.10 | S1PR3^a^-F263L/I284L | 20.07  7.70±0.05 | 84.59 | S1PR1^b^- F133C/V209I/L213I | 1488.00  5.83±0.08 | 77.12 | S1PR1^b^-S129G | 16.02  7.80±0.06 | 92.39 |
| S1PR1^b^ | | 33.09  7.48±0.05 | 100.00 | S1PR1^b^ | 5.51  8.26±0.07 | 100.00 | S1PR1^b^-S129G | 109.70  6.96±0.04 | 89.43 | S1PR1^b^-T207I | 132.60  6.88±0.12 | 118.9 |
| S1PR1^b^- F133C/  V209I/L213I | | 896.6  6.05±0.08 | 125.7 | S1PR1^b^- F133C/V209I/L213I | 144.90  6.84±0.07 | 56.08 | S1PR1^b^-T207I | 167.00  6.78±0.09 | 93.94 | S1PR1^b^-V132T | 31.61  7.50±0.10 | 90.61 |
| S1PR3^b^ | | 2237.00  5.65±0.03 | 100.00 | S1PR3^b^ | 495.80  6.31±0.07 | 100 | S1PR1^b^-V132T | 27.31  7.56±0.02 | 93.38 | S1PR1^b^-F133L/V209A/L213I | 2624  5.58±0.16 | 121.6 |
| S1PR3^b^- C127F/I203V/I207L | | 35.35  7.45±0.08 | 50.22 | S1PR3^b^- C127F/I203V/I207L | 17.14  7.77±0.08 | 140.30 | S1PR3^a^ | 1361.00  5.87±015 | 100.00 | S1PR3^a^ | 4293.00  5.37±0.10 | 100.00 |
| S1PR1^c^ | | 26.7  7.57±0.06 | 100.00 | S1PR1^c^ | 4.45  8.35±0.07 | 100.00 | S1PR3^a^-F263L/I284L | 25.05  7.61±0.08 | 72.18 | S1PR3^a^-F263L/I284L | 87.95  7.06±0.12 | 52.38 |
| S1PR1^c^-R78A | | 38.10  7.42±0.09 | 68.92 | S1PR1^c^-R78A | 182.80  6.74±0.09 | 114.50 | S1PR3^b^ | 1342.00  5.87±0.04 | 100.00 | S1PR3^b^ | 7216.00  5.14±0.13 | 100.00 |
| S1PR1^c^-M124V | | 95.99  7.02±0.07 | 70.38 | S1PR1^c^-M124V | 424.70  6.37±0.07 | 116.20 | S1PR3^b^-G123S | 81.97  7.09±0.03 | 91.68 | S1PR3^b^-G123S | 1315.00  5.88±0.08 | 100.2 |
| S1PR1^c^-S129T | | 249.10  6.60±0.07 | 76.30 | S1PR1^c^-S129T | 401.60  6.40±0.07 | 109.50 | S1PR3^b^-I201T | 621.70  6.21±0.06 | 80.64 | S1PR3^b^-I201T | 749.70  6.13±0.11 | 78.94 |
| S1PR1^d^ | | 23.77  7.62±0.05 | 100.00 | S1PR1^b^-F133L/V209A/L213I | 23.29  7.63±0.09 | 66.27 | S1PR3^b^- C127F/I203V/I207L | 38.30  7.42±0.11 | 57.88 | S1PR3^b^- C127F/I203V/I207L | 6.94  8.16±0.12 | 57.10 |
| S1PR1^d^-F133L/  V209A/  L213I | | 138.60  6.86±0.07 | 85.65 | S1PR1^b^-T207V | - | - | S1PR3^b^-T126V | 1173.00  5.93±0.07 | 78.61 | S1PR3^b^-T126V | 199.00  6.70±0.09 | 59.72 |
| S1PR1^d^-T207V | | 141.00  6.85±0.06 | 109.30 | S1PR5^a^ | 272.00  6.57±0.24 | 155.100 | S1PR1^c^ | 38.09  7.42±0.06 | 100.00 | S1PR1^a^-R78A | 50.98  7.29±0.10 | 96.34 |
| S1PR5^a^ | | 172.50  6.76±0.12 | 100.00 | S1PR5^a^-A69R | 43.85  7.36±0.10 | 115.40 | S1PR1^c^-R78A | 187.70  6.73±0.08 | 80.66 | S1PR1^a^-M124V | 9.95  8.00±0.14 | 71.56 |
| S1PR5^a^-A69R | | 989.80  6.00±0.12 | 110.40 | S1PR5^a^-V115M | 49.07  7.31±0.12 | 100.00 | S1PR1^c^-M124V | 114.40  6.94±0.05 | 110.40 | S1PR1^a^-S129T | 11.18  7.95±0.19 | 55.88 |
| S1PR5^a^-V115M | | 43.59  7.36±0.19 | 78.95 | S1PR5^a^-T120S | 90.73  7.04±015 | 161.10 | S1PR1^c^-S129T | 1011.00  6.00±0.09 | 80.54 | S1PR1^b^ | 6.16  8.21±0.07 | 100.00 |
| S1PR5^a^-T120S | | 319.60  6.50±0.14 | 117.1 | S1PR5^b^ | 991.10  6.00±0.17 | 148.20 | S1PR1^d^ | 22.33  7.65±0.07 | 100.00 | S1PR1^b^- F133L/V209A/L213I | 77.22  7.11±0.07 | 98.31 |
| S1PR5^b^ | | 150.80  6.82±0.08 | 100.00 | S1PR5^b^-V198T | 95.39  7.02±0.07 | 106.5 | S1PR1^d^-T207V | 182.3  6.74±0.11 | 64.66 | S1PR1^b^-T207V | 245.10  6.61±0.08 | 131.1 |
| S1PR5^b^-V198T | | 22.02  7.66±0.10 | 91.44 | S1PR5^b^- L124F/A200V/I204L | 88.96  7.05±0.09 | 100.00 | S1PR1^d^-F133L/V209A/L213I | 65.42  7.18±0.06 | 76.84 | S1PR5^a^ | 78.25  7.11±0.11 | 100.00 |
| S1PR5^b^- L124F/A200V/I204L | | 11.48  7.94±0.08 | 99.96 |  |  |  | S1PR5^a^ | 678.20  6.17±0.26 | 100.00 | S1PR5^a^-A69R | 28.60  7.54±0.31 | 53.12 |
|  | |  |  |  |  |  | S1PR5^a^-A69R | 317.30  6.50±0.11 | 264.6 | S1PR5^a^-V115M | 68.83  7.16±0.33 | 31.59 |
|  | |  |  |  |  |  | S1PR5 ^a^ -V115M | 99.09  7.00±0.08 | 323.40 | S1PR5^a^-T120S | 635.90  6.20±0.17 | 64.80 |
|  | |  |  |  |  |  | S1PR5 ^a^ -T120S | 874.10  6.06±0.13 | 334.1 | S1PR5^b^ | 71.42  7.15±0.10 | 100.00 |
|  | |  |  |  |  |  | S1PR5^b^ | 737.60  6.13±0.09 | 100.00 | S1PR5^b^-V198T | 8.09  8.09±0.13 | 81.92 |
|  | |  |  |  |  |  | S1PR5^b^-V198T | 469.50  6.33±0.15 | 68.39 | S1PR5^b^- L124F/A200V/I204L | 17.36  7.76±0.09 | 97.98 |
|  | |  |  |  |  |  | S1PR5^b^-L124F/A200V/I204L | 148.60  6.83±0.07 | 73.02 |  |  |  |
